# Recycling Waste Circuit Board Efficiently and Environmentally Friendly through Small-Molecule Assisted Dissolution

**DOI:** 10.1038/s41598-019-54045-w

**Published:** 2019-11-29

**Authors:** Zhiqiang Chen, Meng Yang, Qian Shi, Xiao Kuang, H. Jerry Qi, Tiejun Wang

**Affiliations:** 1 State Key Lab for Strength and Vibration of Mechanical Structures, Department of Engineering Mechanics, Xi’an Jiaotong University, Xi’an, 710049 China; 2 The George W. Woodruff School of Mechanical Engineering, Georgia Institute of Technology, Atlanta, GA 30332 USA

**Keywords:** Environmental chemistry, Pollution remediation, Sustainability

## Abstract

With the increasing amount of electronic waste (e-waste) generated globally, it is an enormous challenge to recycle printed circuit boards (PCBs) efficiently and environmentally friendly. However, conventional recycling technologies have low efficiency and require tough treatment such as high temperature (>200 °C) and high pressure. In this paper, a small-molecule assisted approach based on dynamic reaction was proposed to dissolve thermosetting polymers containing ester groups and recycle electronic components from PCBs. This effective approach operates below 200 °C and the polymer could be dissolved in a short time. It has a remarkable ability to recycle a wide range of commercial PCBs, including boards made of typical anhydride epoxy or polyester substrate. Besides, it is environmentally friendly as even the recycling solution could be reused multiple times. In addition, the wasted solution after recycling could be used for board bonding and damage repair. This work also demonstrates the advantage of using polymers containing ester groups as the PCB substrate in consideration of eco-friendly and efficient recycling.

## Introduction

With the rapid development of the economy, electronic products, such as personal computers, mobile phones, control equipment, and machines, are ubiquitous in modern society. Due to the increasing usage of electronic products, the total amount of electronic waste (e-waste) generated globally has continued to grow at a rate of 3–5% annually and is expected to be approximately 50 million tons (Mt) in 2018^1,2^. How to manage the e-waste has become a hotly debated issue in the 21st century^3,4^. To achieve a sustainable economy, it is necessary to develop innovative green chemistries towards the end-of-life recovery of used products and reuse of recovered materials^5,6^.

Printed circuit boards (PCBs), the integral part of any electronic products, accounts for a great percentage of the total weight among those typical components dismantled from the e-waste. On one hand, waste PCBs contain heavy metal elements, organic matters (such as resin and brominated flame retardants) and chemical residuals that may cause a serious threat to the environment as well as human health^7,8^. On the other hand, waste PCBs have great residual value due to the presence of high-grade precious metals (~28% weight) such as Au, Ag, Cu, Pd, Ta and so on. In addition, the metallic grade in PCBs is more than a hundred times of that in natural mineral resources^9,10^. Therefore, recycling waste PCBs plays a critical role in both environmental protection and economic development.

PCBs are commonly composed of fiberglass reinforced epoxy composites, electronic components, and various additives. The most critical step in recycling PCBs is to separate electronic components from the composites through removing or degrading organic materials. In addition, it is better to recycle the fiberglass and other materials like epoxy resin for the further reuse. To date, several recycling technologies using mechanical, chemical and thermal approaches have been investigated^11–19^. In traditional mechanical recycling methods, waste PCBs are selectively dismantled and crushed and then physical separation using magnetic or electrostatic methods is used to obtain various metal particles. The non-metallic fraction from waste PCBs could also be added as fillers to fabricate high-strength composites^20,21^. This approach is relatively low cost and environment-benign, making it widely used in many countries. However, it is also inefficient due to the loss of metal during separation. In contrast, thermal or chemical recycling technologies can remove non-metallic materials (organic substrate or plastics) and obtain purified metals from metallic powders through pyrometallurgical or hydrometallurgical processes. Therefore, these methods enjoy much higher efficiency and economical return. However, these methods typically require relatively high processing temperature (>200 °C) or high pressure. In addition, the exhaust hazardous fumes and strong chemicals (like acid/alkali) in the primitive recycling process may lead to serious environmental pollution^17,22^. Although significant efforts have been invested, efficiently and environmentally friendly recycling PCBs still remains an enormous challenge. One critical issue is how to remove organic materials, which are chemically cross-linked and are designed to be intrinsically stable. To address this issue, research has been conducted to seek alternative matrix material for PCBs^23–28^. For example, paper-based electronics have been reported to show advantages over traditional organic PCBs in terms of their environmental impacts. However, they are only applicable to some special fields due to their intrinsic drawbacks, such as reliability in the presence of humidity.

In the recent decades, new explorations of dynamic covalent chemistry have opened the door to recycle thermosetting polymers and composites under relatively mild conditions through bond exchange reactions, such as transesterification reactions^29,30^, thiol-disulfide exchange reactions^31,32^, Diels-Alder reactions^33^, among many others^34–37^. However, these thermoset resins need complex synthesis procedures and the dynamic nature of the network would induce dramatic drops in mechanical performance at high temperatures above 160 °C, which severely restricts their applications in industrial practice. Recently, our group developed a new approach to dissolve anhydride epoxy, which is one of typical thermoset polymers used in industry^38,39^, through transesterification reaction between the hydroxyl group in ethylene glycol (EG) solvent and the ester bond in epoxy network. In this paper, we attempted to use this small-molecule assisted method to recycle waste PCBs. The solvent composed of EG, catalyst and organic solvent was used to dissolve the thermoset substrate containing ester groups in waste PCBs, and then the electronic components could be easily separated from circuit boards. Our work shows the small-molecule assisted method has a remarkable ability to recycle a wide range of commercial PCBs, including boards made of typical epoxy-anhydride and polyester resin substrate. Besides, those chemicals in this method can be recovered for reuse. The wasted solution after recycling procedure was also shown to have potential value in multilayer board fabrication and damage repair. These demonstrate the potential of using polymer containing ester groups as board substrate to replace widely used other industrial materials in consideration of eco-friendly and efficient recycling.

## Experimental

### Materials

The monomers diglycidyl ether of bisphenol A (DGEBA, MW: 340.41 g/mol), glutaric anhydride (GA, MW: 114.10 g/mol), and 1,5,7-Triazabicyclo[4.4.0]dec-5-ene (TBD, MW: 139.20 g/mol) were purchased from Sigma Aldrich (St. Louis, MO, USA) and used as received without further purification. Methyl tetrahydrophthalic anhydride (MTHPA, MW: 166.17 g/mol) was obtained from Jining Huakai Resin Co. (Shandong, China). Ethylene glycol (EG) and N-Methyl-2-Pyrrolidinone (NMP) were ordered from Adamas Reagent Co., Ltd (Shanghai, China). 2,4,6-Tris(dimethylaminomethyl)phenol (DMP-30, MW: 265.39 g/mol; Shanghai Macklin Biochemical Co., Ltd, China) was selected as the accelerant to fabricate pure epoxy resin. The chemical structures of above monomers are shown in Fig. S1 (Supporting Information). Iron (III) chloride (Shanghai Aladdin Bio-Chem Technology Co., Ltd) was used to etch the electronic circuits. The commonly used type 7628 glass fabric in the electronics industry was obtained from Feipufu Co. (Beijing, China) to fabricate composites. The plain weave fiberglass cloth has a thickness of ~0.20 mm.

### Preparation of composite and circuit board

The manual impregnation method was used to prepare the composite materials in this work. Epoxy resin was firstly prepared as follows: DGEBA and MTHPA (or GA) in solid-state were melted under 100 °C and then DMP-30 (weight ratio 0.02) was added to promote the solidification process. The mole ratio between the epoxy group and the acyl group was 1:1. The glutinous liquid was mixed together at a stirring speed of ~300 rpm (Magnetic stirrer, IKA C-MAG HS7, IKA Works Inc., Wilmington, NC, USA) and then placed in a vacuum chamber to remove the remaining bubbles.

To prepare the composites, an 80 mm × 100 mm glass mold with a specified thickness was prepared and covered with Teflon tape, which was used to prevent epoxy resin from bonding with glass during the curing process. A fiberglass sheet was placed on the mold and was manually impregnated using the mixture obtained in the first step. Then, other layers of glass fiber were placed and the same operation was repeated. After the manual impregnation, the mold was sealed by a Teflon covered glass plate (80 mm × 100 mm) and a 2 kg weight was placed on top to excrete excess resin and ensure the dimensional accuracy. Lastly, curing process was carried out thoroughly in an oven at 80 °C for 2 hours and at 130 °C for additional 4 hours. After being cooled to room temperature, multilayered epoxy composites (4 layers, total thickness = 2 mm) were prepared for the following experiments.

To make circuit boards in the lab, a copper foil was firstly used to cover the composite (40 mm × 30 mm) and then an oily pen was used to draw electric lines as designed. Iron(III) chloride solution (500 g/L) was used to etch away exposed copper at 50 °C for 20 min. Then, the oily ink was cleaned by alcohol and the desired electronic circuits were obtained. The etching process could be seen in Fig. S2. Lastly, electronic components, such as LED, resistors, and capacitors, were soldered onto the circuit board by a welding gun and soldering tin. In addition, a commercial PCB was purchased from Telesky Electronics Co. (Shenzhen, China). It was fabricated as 315 M wireless receiving modules. The flexible polyester PCBs were obtained from Qixin Weiye Circuit Co. (Shenzhen, China). The polyester substrate was made of polyethylene terephthalate (PET).

### Pre-polymerized adhesive and board bonding

The pre-polymerized adhesive was prepared for board bonding in Section 3.3 as follows. Firstly, catalyst TBD (mole ratio: 0.02) was added into mixed DGEBA and glutaric anhydride. The mixture was then stirred at 130 °C for 3~10 min until the transparent viscous liquid with yellowish color was obtained. After removing the remaining bubbles, the adhesive solution composed of catalysts, oligomers, and monomers was ready for study.

To bond composite boards using pre-polymerized adhesive, the composite surfaces were cleaned by alcohol without any other surface modification. Next, the pre-prepared adhesive was evenly smeared onto the laminates (30mmx15mm) and then the specimen was pressed lightly to remove the excessive glue. After heating at 130 °C for 30 min, the two composite boards were bonded together to form a reliable joint. Finally, single lap joint samples with an overlapping area of 15 mm × 15 mm were assembled for the lap shear tests, which were conducted at a static loading speed of 13 mm/min to obtain the bonding strength.

## Results and Discussion

### Recycling mechanism and application in composites

**Figure 1:**
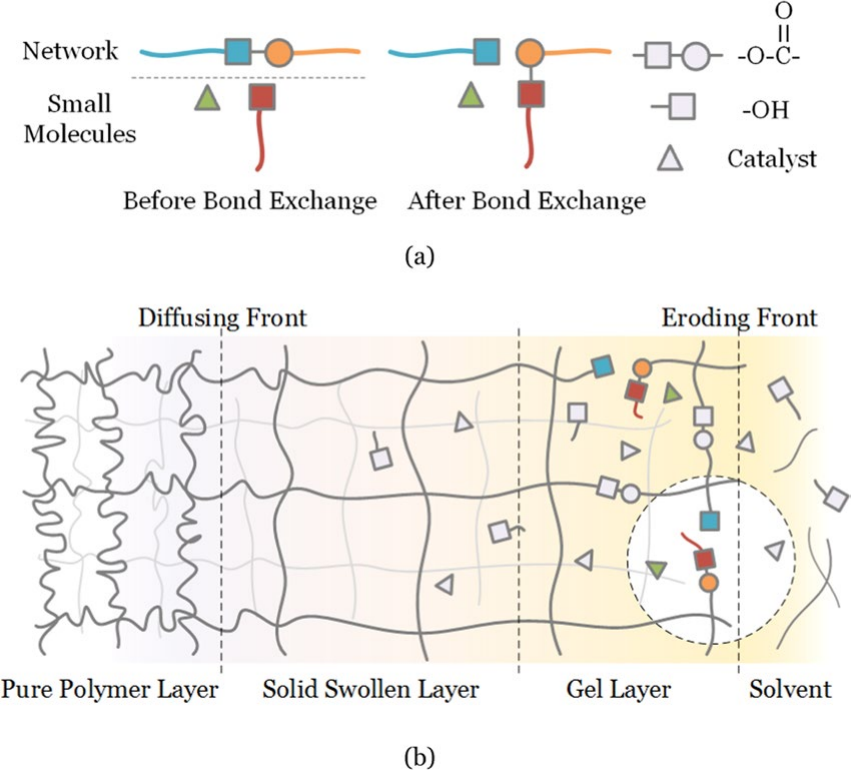
(**a**) The process of bond exchange reaction via transesterification. Hydroxy groups in solvent break ester bonds in the polymer network. (**b**) Mechanism of the small-molecule assisted dissolution method.

The degradation of organic materials is the key step in recycling waste PCBs. The mechanism of the small-molecule assisted dissolution method is shown in Fig. 1. The recycling solvent contains ethylene glycol (EG), organic solvent (NMP) and transesterification catalyst (TBD) with a mole ratio of 1:1:0.02. Figure 1a illustrates the transesterification-type bond exchange reaction between hydroxy groups in the solvent and ester groups in the polymer network. Ester bonds in the polymer network are cleaved by EG, which leads to the dissolution of the thermoset substrate into oligomers and monomers. The thermosetting polymer dissolution kinetics can be characterized by a surface layer model containing three layers, namely the gel layer, the solid swollen layer, and the pure polymer layer^39^ (Fig. 1b). Diffusion of organic solvent (NMP) makes the thermosetting resin swell quickly, allowing abundant EG and catalyst to enter into the polymer network. A thicker gel layer is formed near the surface layer where the bond exchange reaction occurs actively and the resin surface is eroded gradually.

**Figure 2:**
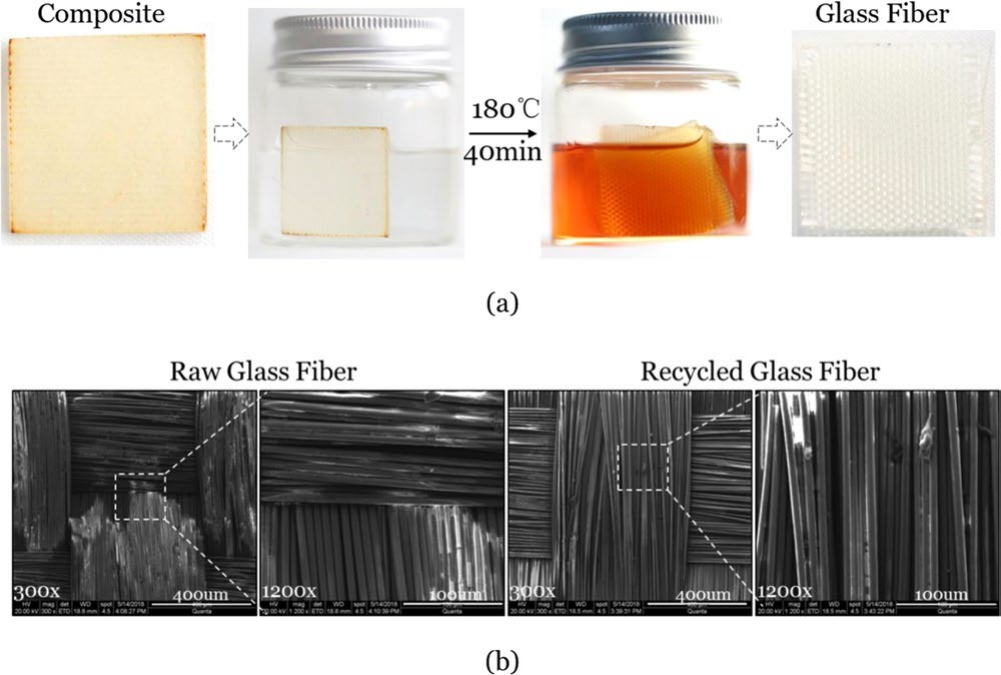
(**a**) Recycling composite board through the small-molecule assisted approach. The composite matrix was MTHPA cured epoxy. (**b**) The SEM micrographs of the raw glass fiber and the recycled glass fiber.

Figure 2 shows the dissolution of the epoxy-fiberglass composites. The composite sample had the dimension of 20 mm(L) × 20 mm(W) × 2 mm(T). We continually checked the degree of dissolution every 5 minutes until the epoxy appeared to disappear. We then used SEM to confirm that there was no residual epoxy resin on the fiberglass. Only 40 min and 2 h were needed to dissolve the composites when heating at 180 °C and 130 °C, respectively. At 100 °C, it needed more than 6 h, which was expected as the temperature was not high enough to trigger sufficient bond exchange reactions. The SEM micrographs of the raw glass fibers and the recycled glass fibers are shown in Fig. 2b. It can be seen that all epoxy was fully depolymerized and the glass fiber bundles appeared to be intact and similar to the raw glass fiber even after a long-time soaking in solution. This enabled the glass fiber to retain its mechanical performance at near-pristine levels. While in traditional recycle technologies, glass fiber was damaged by mechanical, thermal or chemical treatment and can only be used for low-grade block materials for construction. It should be noted that, compared with complete dissolution based on catalyst participating transesterification, no depolymerization of MTHPA cured epoxy was observed in the EG-NMP solution without catalyst even after heating at 160 °C for 3 h (Fig. S3). There were only a few of epoxy particles floating in solution, which were peeled from the composite surface due to thermal stress and swelling force. In our experiments, the time (40 min, 180 °C) to completely dissolve the composite was similar to that of pure epoxy with the same dimension (20 mm × 20 mm × 2 mm), which was also 40 min. Overall, the presence of fiberglass did not reduce the efficiency of epoxy dissolution.

### Recycling circuit boards

The waste PCB recycle system through the small-molecule assisted approach has two simple steps: first, the recycling solution dissolves the thermoset substrate of the waste PCB; second, the electronic components including circuits, resistors, and capacitors etc. are separated from the circuit boards. It should be noted that it is common in the electronic industry to mount most of the electronic elements on the circuit boards directly, which makes the separation step very easy once the thermoset substrate is dissolved. Actually, some researches have reported that organic solvents could be utilized to break the bonding force (internal van der Waals bonds) between bromine epoxy resin and other materials, which leads to components separation from circuit board subsequently^40–43^. However, this technique cannot depolymerize thermosetting networks and requires complex treatment. Thus, it is difficult to achieve an efficient material recovery, especially for glass fibers and electronic components encapsulated in thermoset resins.

#### Recycling in-house made PCB

**Figure 3:**
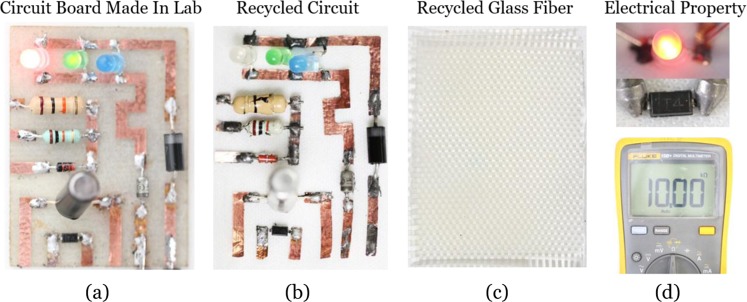
(**a**) The circuit board made in the lab. The substrate was the anhydride-epoxy composite. (**b**) The recycled circuit. (**c**) The recycled glass fiber. (**d**) Electrical property of recycled electronic components.

Figure 3 shows the lab-made circuit board and the recycled components. As shown in Fig. 3b,c, both circuit elements and glass fiber were fully recycled with our approach after heating at 180 °C for 40 min. Besides, although the plastics that covered the resistors or the capacitors were also dissolved, the electrical properties of these electronic components were preserved (Fig. 3d). Therefore, majorities of the electronic components could be used again within their service life if these components could be resealed. Therefore, the small-molecule assisted recycling strategy has the advantage of resource saving while traditional technologies always destroy the physical structure of circuit elements or need tedious disassembly to retain valuable materials in pre-preparation. Once the electronic components are harvested through the above-mentioned procedures, the pure high-grade metals could also be recovered subsequently through the existing purification technologies such as pyrometallurgy or hydrometallurgy.

#### Recycling commercial epoxy PCB

**Figure 4:**
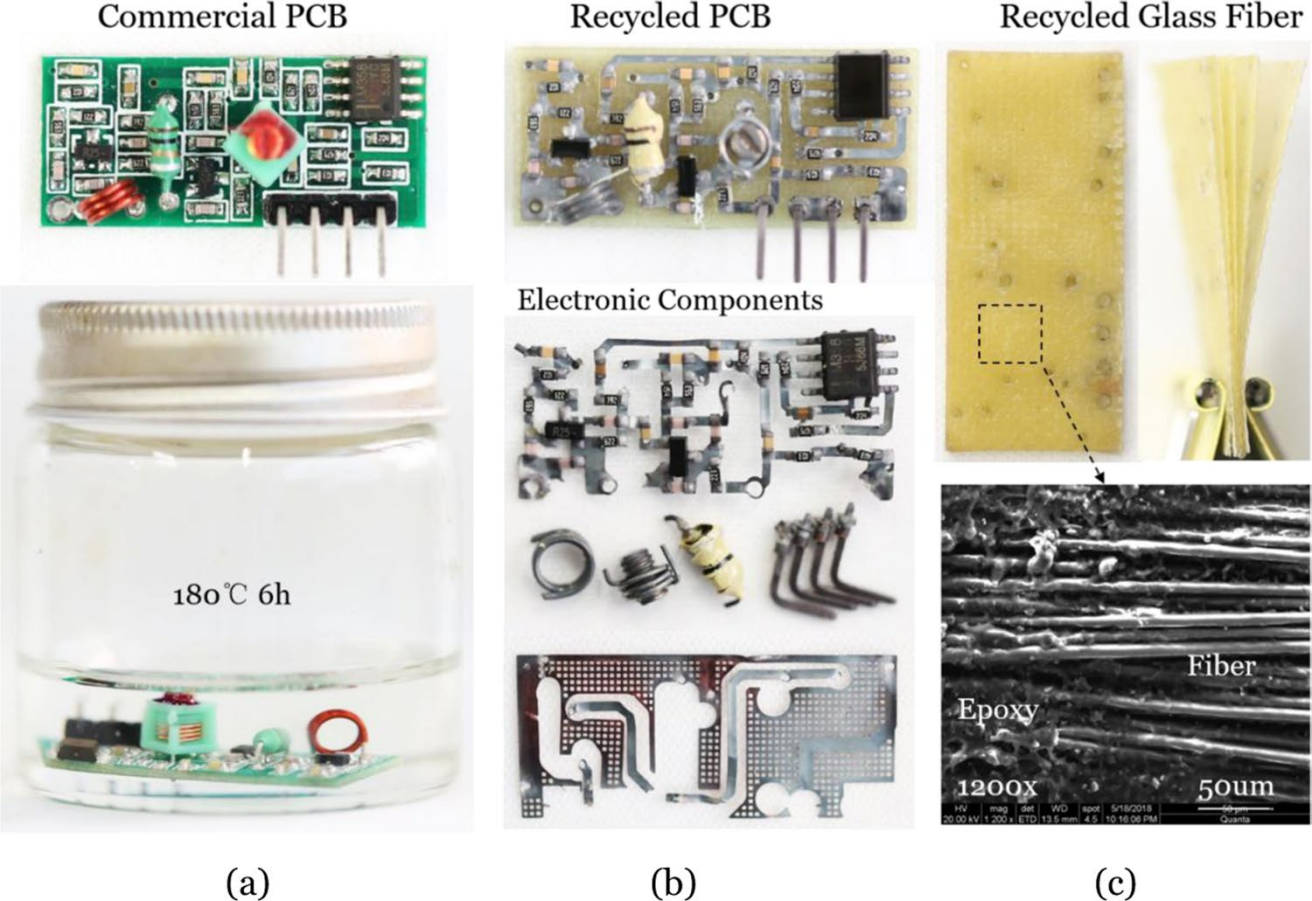
Recycling commercial epoxy PCB through the small-molecule assisted approach. (**a**) The commercial PCB sample and the recycling treatment. (**b**) The recycled circuit and the electronic components. (**c**) The recycled glass fiber and the SEM micrograph.

Figure 4 illustrates the recycling process of the commercial epoxy PCB with the small-molecule assisted approach. By heating at 180 °C for 6 h, sufficient bond exchange reactions were triggered. As a result, thermosetting polymer substrate degraded gradually with deep penetration of small molecules. Plastics with low melting point were also dissolved at elevated temperatures. After heating, the transparent solution became green due to the dissolution of the solder resist ink on the surface. The PCB after the dissolution procedure was then taken out and cleaned with alcohol. Due to the depolymerization of the surface polymer, it was quite easy to peel off the whole circuit from the substrate board while only a few elements that penetrated through the PCB needed manual resection with a pair of pliers (Fig. 4b). We found that most of the electronic components retained their original electronic properties. In addition, comparing to traditional recycling methods that destroy fibers, the layered fiber cloth could be well obtained in our recycle method. In Fig. 4c, the SEM image of the recycled glass fiber cloth shows exposed fiber bundles and some residual polymer. This proved that most of the epoxy matrix was decomposed by the small-molecule assisted dissolution. As some other kinds of agents were usually added to make industrial boards, there was a small amount of residual polymer after the dissolution. In the electronic industry, epoxy resin is a common candidate as adhesive to fabricate multilayer circuit boards. Thus, even the composites are made of any other kinds of polymer, our small-molecule assisted approach may have the ability to separate the circuits from the board through the dissolution of bonding layer as long as the adhesive epoxy layer contains ester groups in the network.

#### Recycling flexible polyester PCB

**Figure 5:**
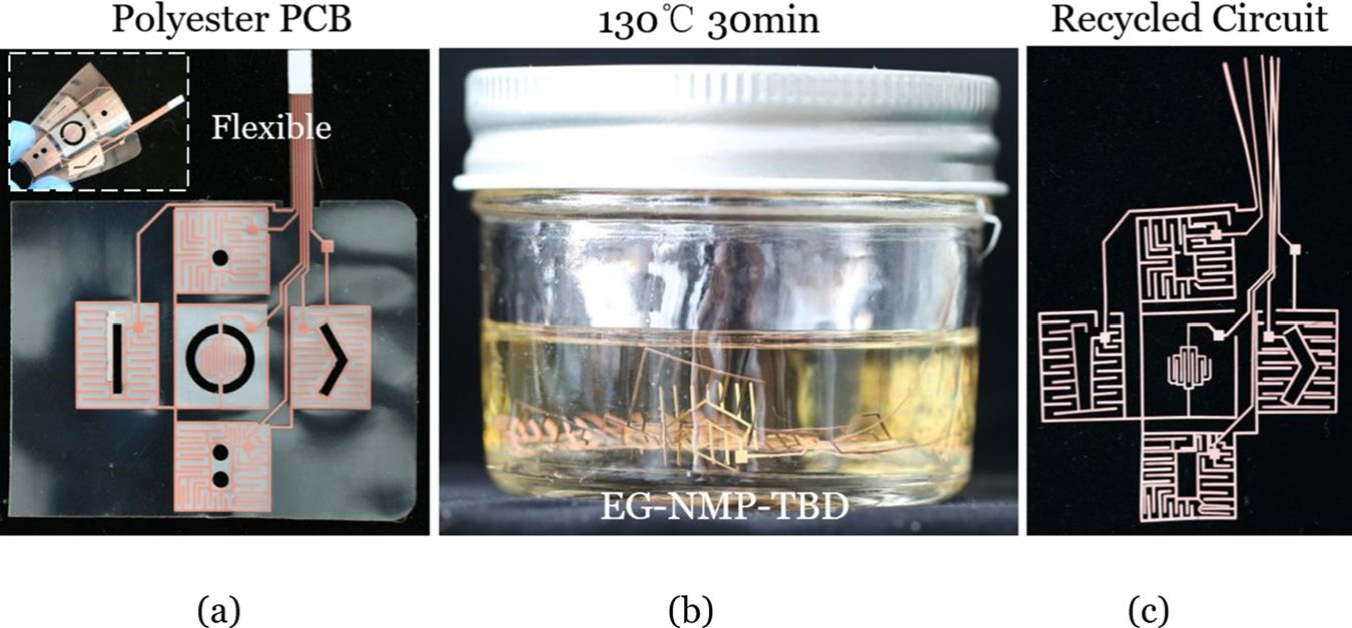
Recycling flexible polyester PCB through the small-molecule assisted approach. (**a**) The polyester PCB sample; the inset shows its good flexibility. (**b**) The degradation process of the polyester matrix. (**c**) The recycled circuit.

In flexible printed circuit boards, polyester and polyimide (market share: about 20% and 80% respectively) are usually selected as the soft substrate^44^. Comparing to polyimide that is thermal-stable but expensive, polyester has advantages such as low cost and transparency and can satisfy most of the general requirements at a mild temperature below 200 °C^45^. As a polyester network contains abundant ester groups, it can be fully degraded through our small-molecule assisted approach. Figure 5 illustrates the application in recycling flexible polyester PCB whose substrate was made of polyethylene terephthalate (PET, melting point: 250–255 °C). As seen in Fig. S4, this type of polyester PCBs was stable and no melting or depolymerization was observed in the EG-NMP solution without catalyst even after heating at 130 °C for a long time (>5 h). However, the PET substrate was completely degraded within 30 min at 130 °C in the recycling solution containing transesterification catalyst (TBD) and the circuits were separated automatically (Fig. 5c). Comparing with other chemical recycling approaches such as using ionic liquids (180 °C, >6 h) or hydrolysis (usually >190 °C, >2 h)^46–48^, the small-molecule assisted dissolution approach greatly simplifies the number of steps and saves time at a lower temperature to degrade pure PET.

Our work demonstrated that anhydride cured epoxy and polyester, due to the ester group in their chemical compositions, can allow bond exchange reactions. In addition, there are several kinds of new ester-containing materials such as latent epoxy and poly(ester-imide)s (PEIs) designed for industrial circuit boards^49–52^. For example, PEIs have been developed as an important class of thermally stable copolymers for heat resistant films and flexible printed circuit boards in recent decades^51,52^. Therefore, the small-molecule assisted approach has the appealing potential in recycling a wide range of commercial circuit boards as long as the substrate polymer network contains ester groups.

### Intrinsic bonding for multilayer board

**Figure 6:**
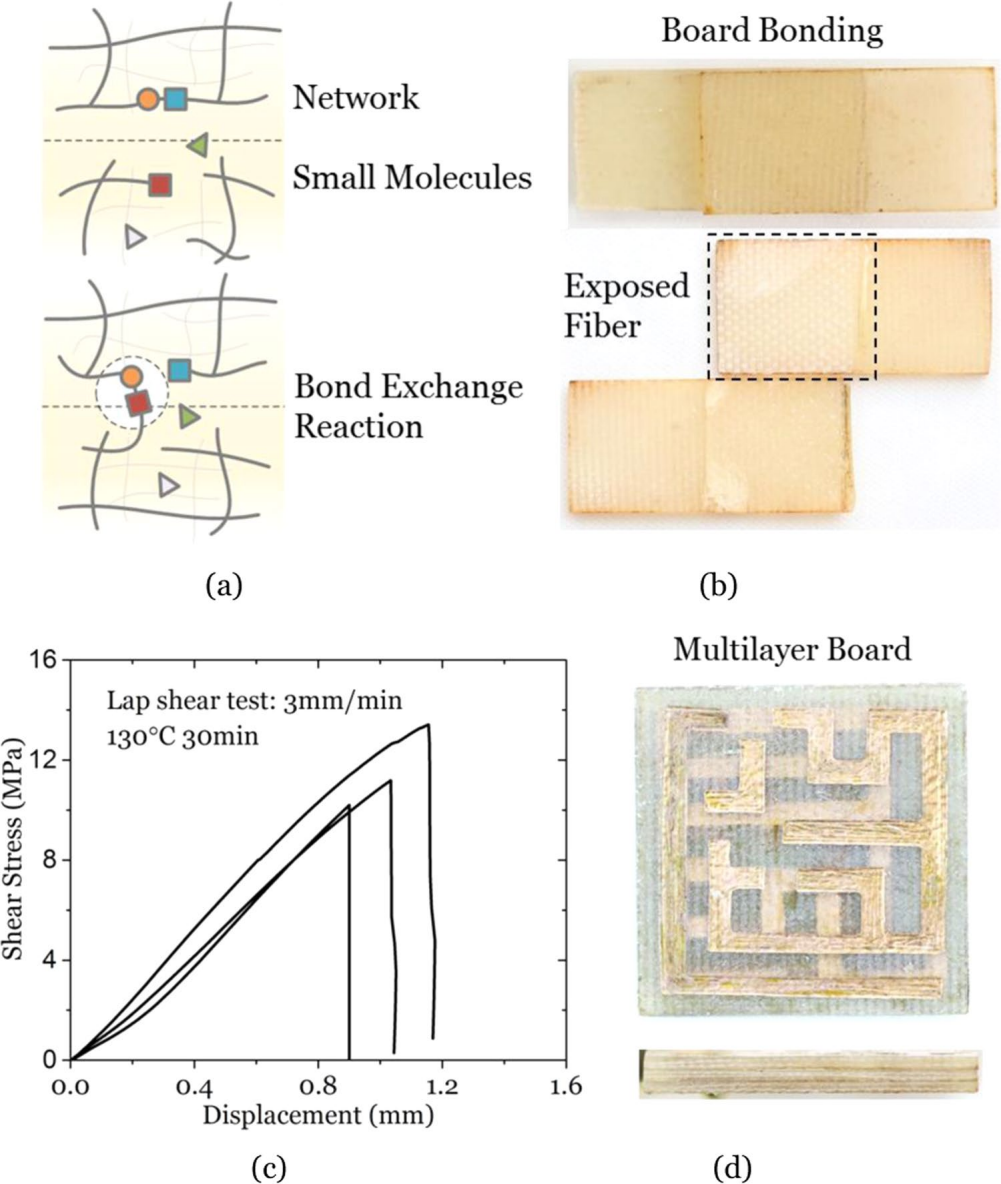
The multilayer board fabrication by using the intrinsic bonding approach. (**a**) The mechanism of intrinsic bonding through bond exchange reaction between the small molecules in the adhesive solution and the polymer network in the composite surface. (**b**) Board bonding sample and failure mode in the lap shear test. The composite matrix was GA-cured epoxy. (**c**) Displacement-shear stress curves of the lap shear tests. (**d**) Multilayer circuit board bonded by four laminates.

The waste solution after recycling procedure could be reused multiple times because of the large content of catalyst and EG. There was virtually no loss in the dissolution efficiency within five times. Certainly, more and more oligomers and monomers also remained in the solution, which may eventually reduce the dissolution efficiency. But the maximum number of actual usages depends on the amount of dissolved molecules. Herein, we propose an intrinsic bonding method to reuse the waste solutions in multilayer board fabrication and damage repair. After removing EG (boiling point: 197 °C) and organic solvent NMP (boiling point: 203 °C), the residual solution contained catalyst, oligomers, and monomers, which had the ability to join boards. Figure 6a demonstrates the intrinsic bonding mechanism. At elevated temperatures, bond exchange reactions between polymer networks and small molecules in the residual solution occurred around the interface. At the same time, oligomers and monomers were re-polymerized. Different from the traditional bonding methods based on weak intermolecular forces, physical interpenetration or mechanical interlock^53,54^, strong covalent bonds were built synergistically to join thermoset composite boards with high-strength interface-phase layer.

Due to the restrictions on the experimental conditions to remove NMP by reduced pressure distillation, we introduced the pre-polymerized adhesive solution to take place of the waste solution with EG and NMP removed. The preparation procedures of pre-polymerized adhesive were shown in Section 2.3. It should be noted that oligomers and monomers in the wasted recycle solution contained short branched chains due to transesterification with EG, thus the pre-polymerized adhesive solution should have similar chemical components of the waste solution. Dimethylformamide (DMF) is also a potential choice instead of NMP for the preparation of the recycling solvent. Firstly, it is easier to evaporate DMF than NMP due to its lower boiling point of 153 °C. Secondly, it is better to use DMF considering the environmental impact. However, at high temperatures (>153 °C), boiling DMF might cause potential danger (like vapor explosion) due to the increasing pressure in the vessel, especially in the lab. Therefore, we did not use DMF in this work. But DMF can be considered in the future industrial applications.

Figure 6b shows the sample after bonding and the failure mode in the lap shear test. With sufficient bond exchange reactions carried out in the vicinity of the interface, the adhesive layer and the composite surface were bonded, forming a robust layer of epoxy cross-linked by covalent bonds. In the lap shear test, the adhesive layer stayed intact while the composite surface epoxy was torn and the exposed fiber cloth was observed clearly. This phenomenon indicated that the fiber-epoxy interface strength was weaker than the adhesive-epoxy interface strength. From the displacement-shear stress curves of the lap shear tests, the shear strength at approximately 10 MPa was obtained (Fig. 6c). Although the highest bonding strength was limited to the weak fiber-epoxy interface strength of the composites used in this work, we believe that bonding strength more than 10 MPa should be attained if using better quality composites. The intrinsic bonding method can be used for gluing individual PCBs into a multilayer circuit board. Figure 6d shows a multilayer circuit board bonded by four composite laminates. The interface between neighboring laminates almost disappeared and the circuits were well packed inside the board. Therefore, the intrinsic bonding method may have a great potential in overcoming the interfacial failure problem in multilayer circuit boards as well as in composite manufacturing.

In addition to strong intrinsic bonding in the multilayer board fabrication, the wasted recycled solution has the potential to repair composite surface damage (Fig. S5). It should be pointed out that this novel bonding and repairing approach through dynamic reactions grants appealing promise in industrial composite applications. The results will be reported in the near future.

## Conclusion

In summary, we proposed a small-molecule assisted method based on dynamic reaction to recycle waste circuit boards effectively and environmentally friendly. Thermoset resins containing ester groups in waste circuit boards were efficiently dissolved through the transesterification reaction at temperatures below 200 °C. Subsequently, the electronic components with reserved electronic properties could be readily separated from the circuit boards. Experimental results showed that this approach had an appealing capability to recycle a wide range of commercial PCBs, including boards made of typical epoxy-anhydride and polyester resin substrate. The concept can also be extended to other types of substrate polymers containing ester groups. This also suggests that introducing ester groups into the substrate polymer network may be a promising route for efficiently and eco-friendly recycling. More notably, the wasted solution after recycling procedure was proved to have the potential value in the multilayer board fabrication. Adhesive solution containing similar chemical components with the waste solution after removing EG and organic solvent was used to join boards by triggering the bond exchange reactions between polymer networks. This intrinsic bonding strategy has a great potential for practical industrial applications, especially in composites.

## Supplementary information

Supplementary information

## Data Availability

All data generated or analyzed during this study are included in this published article.
