# Supplementary material for: Recycling Waste Circuit Board Efficiently and Environmentally Friendly through Small-Molecule Assisted Dissolution
Source: Sci Rep. 2019 Nov 29;9:17902. doi: 10.1038/s41598-019-54045-w (PMC6884634; doi:10.1038/s41598-019-54045-w)
Supplement: Supplementary file 1 — Supplementary information [file 41598_2019_54045_MOESM1_ESM.pdf]

Supporting Information

Recycling Waste Circuit Board Efficiently and Environmentally Friendly through Small-Molecule Assisted Dissolution

Zhiqiang Chen[a], Meng Yang[a], Qian Shi[a], Xiao Kuang[b], H. Jerry Qi[b, *], Tiejun Wang[a, *]

[a] State Key Lab for Strength and Vibration of Mechanical Structures, Department of Engineering Mechanics, Xi'an Jiaotong University, Xi'an 710049, China

[b] The George W. Woodruff School of Mechanical Engineering, Georgia Institute of Technology, Atlanta, GA 30332, USA

* Corresponding authors: qih@me.gatech.edu (H. Jerry Qi), wangtj@xjtu.edu.cn (Tiejun Wang)

Table of Supporting Information

1. Chemical structure view of the reagents used in this study.
2. The etching process to make electronic circuits in lab.
3. Composite in EG-NMP (mole ratio=1:1) solution.
4. Polyester PCB in EG-NMP (mole ratio=1:1) solution.
5. Composite surface damage repair.

1. **Chemical structure view of the reagents used in this study**

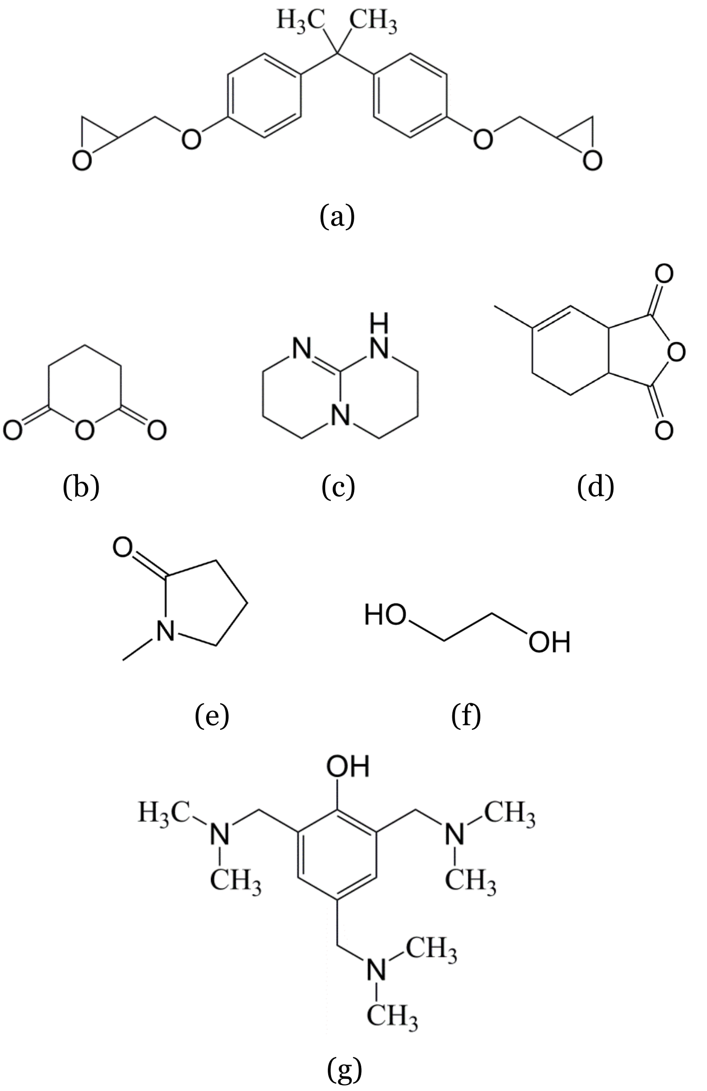

Figure S1. Chemical structure of the reagents used in this study. (a) Epoxy monomer diglycidyl ether of bisphenol A (DGEBA). (b) Curing agent glutaric anhydride. (c) Catalyst 1,5,7-Triazabicyclo[4.4.0]dec-5-ene (TBD). (d) Methyl tetrahydrophthalic anhydride (MTHPA). (e) N-Methyl-2-Pyrrolidinone (NMP). (f) Ethylene glycol (EG). (g) Accelerant 2,4,6-Tris(dimethylaminomethyl)phenol (DMP-30).

2. The etching process to make electronic circuits in lab

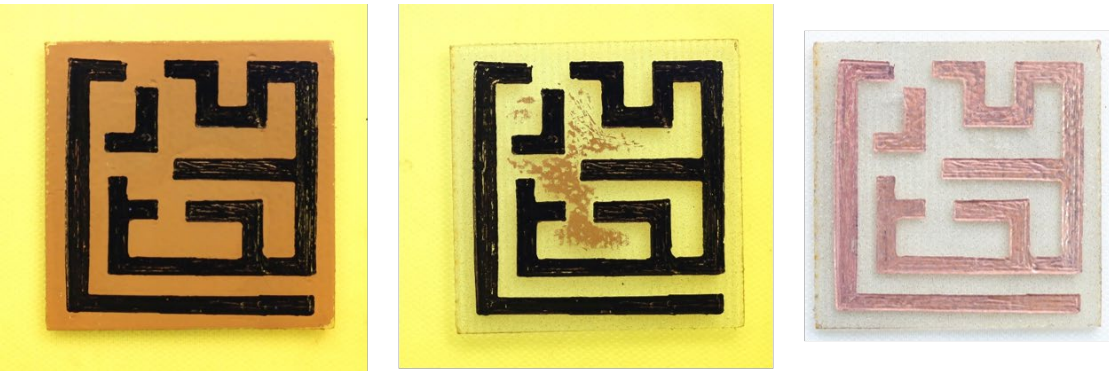

(a) (b) (c)

Figure S2. The etching process to make electronic circuits in lab. The yellow color was Iron(III) chloride solution. (a) Copper covered board with designed electronic lines drawn by oily pen. (b) Iron(III) chloride etched away almost all exposed copper. (c) Board with desired electronic circuit was obtained.

3. Composite in EG-NMP (mole ratio=1:1) solution

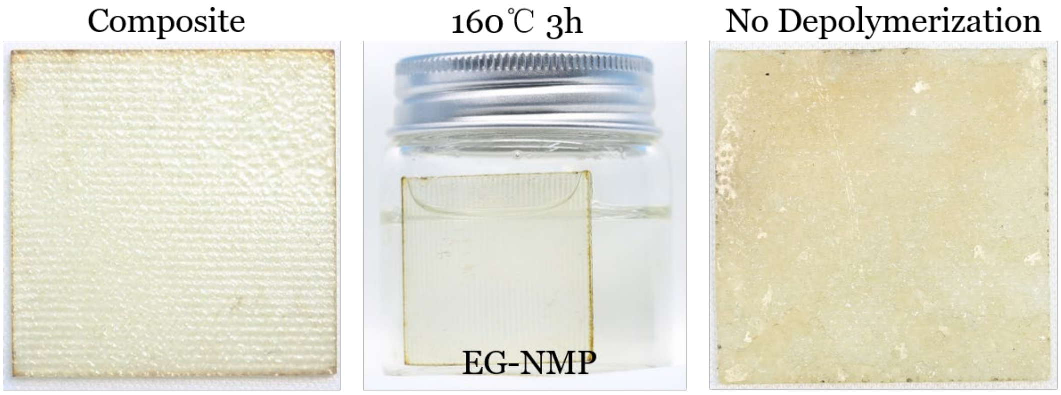

(a) (b) (c)

Figure S3. Composite in EG-NMP (mole ratio=1:1) solution. (a) Composite sample. The matrix was MTHPA cured epoxy. (b) Heating at 160°C for 3h. (c) No depolymerization was observed during heating process. A few epoxy particles were peeled from composite surface due to thermal stress and swelling force.

4. **Polyester PCB in EG-NMP (mole ratio=1:1) solution**

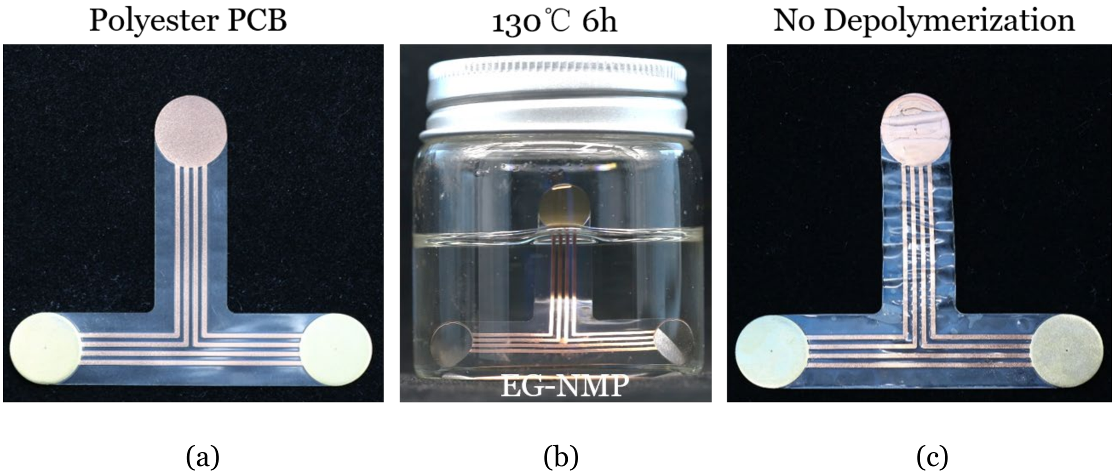

Figure S4. Polyester PCB in EG-NMP (mole ratio=1:1) solution. (a) Polyester PCB sample. (b) Heating at 130°C for 6h. (c) No depolymerization on polyester substrate. Due to thermal stress and swelling force, wrinkle was observed on the edge of material.

5. **Composite surface damage repair.**

Apart from strong intrinsic bonding in multilayer board fabrication, the wasted recycled solution also has a great capability in repairing composite surface damage. The repairing procedure is illustrated in Figure S5. Initially, a fresh composite (matrix resin: GA cured epoxy) was manually scratched on the polymer surface to render a damage area. Then the damaged surface was covered by adhesive solution. At elevated temperature, sufficient bond exchange reactions at the adhesive-polymer interface were triggered during the curing process. As a result, the scratch disappeared completely after healing at 130°C for 30min. Although pre-polymerized adhesive solution used here is not exactly the same as wasted recycled solution, we believe that the wasted recycled solution has potential value in damage repair.

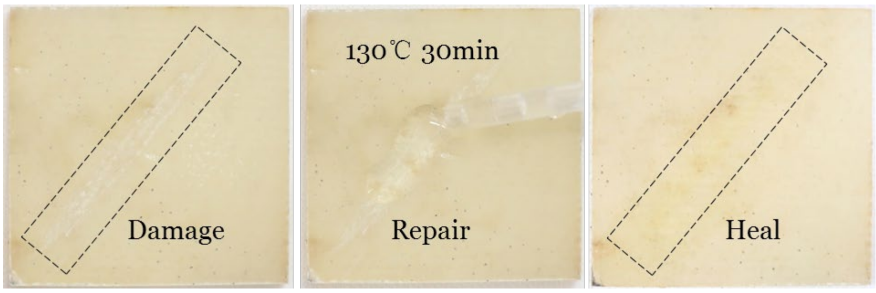

Figure.S5 Repairing composite board with surface damage. Cover the scratch with the adhesive solution. After healing at 130°C for 30min, the scratch disappeared completely.
